# Edward James Sheppard

**Published:** 1915-12

**Authors:** 


### EDWARD JAMES SHEPPARD, L.R.C.P., L.R.C.S. Ed., AND L.M.

Edward James Sheppard was born at Warbleton, in Sussex, on July 17th, 1845. His early education was at Wiesbaden and Heidelberg; he then went to King's College, and afterwards to the Agricultural College at Cirencester.

He married, in 1870, Miss S. D. Jones, and took a farm near Kingsteignton, Devonshire.

He entered at the Bristol Medical School when he was over thirty years of age. He then went to Edinburgh and got qualified in 1884.

Besides his work as a general practitioner, he was Public Vaccinator for the Clifton District, and he attended gratuitously, for more than twenty-five years, the inmates of St. Joseph's Home for the aged and infirm poor.

Some years after the death of his first wife he married, in 1904, Miss Mary Mountfort Barnes.

Whilst cycling down Maudlin Street on September 18th of this year he was knocked down by a motor bus, and sustained severe fractures of the arm and pelvis. He was taken to the Bristol Royal Infirmary, where he died from his injuries on September 20th.

Sheppard was a tall, good-looking man, always honourable and reliable, and a well-known and much esteemed practitioner.

